# Genome Sequence of the Fungus Nannizziopsis barbatae, an Emerging Reptile Pathogen

**DOI:** 10.1128/MRA.01213-20

**Published:** 2021-01-07

**Authors:** Daniel Powell, Ashley Jones, Nicola Kent, Parwinder Kaur, Ido Bar, Benjamin Schwessinger, Céline H. Frère

**Affiliations:** a Global Change Ecology Research Group, School of Science, Technology, and Engineering, University of the Sunshine Coast, Sippy Downs, QLD, Australia; b Research School of Biology, The Australian National University, Acton, Canberra, ACT, Australia; c School of Agriculture and Environment, Faculty of Science, University of Western Australia, Perth, WA, Australia; d Environmental Futures Research Institute, School of Environment and Sciences, Griffith University, Nathan, QLD, Australia; Vanderbilt University

## Abstract

Nannizziopsis barbatae is an emerging fungal pathogen capable of causing contagious dermatomycosis in reptiles. Here, we report a 31.54-Mb draft genome sequence of an isolate originating from an infected eastern water dragon in Brisbane, Australia.

## Abstract (precis)

Nannizziopsis barbata is an emerging fungal pathogen capable of causing contagious dermatomycosis in reptiles. Here, we report a 31.54-Mb draft genome sequence of an isolate originating from an infected eastern water dragon in Brisbane, Australia.

## ANNOUNCEMENT

Nannizziopsis barbatae is a keratinophilic ascomycetous fungus of the family *Onygenaceae*. Recently distinguished from the *Chrysosporium* anamorph of Nannizziopsis vriesii (CANV) complex, this species was originally isolated from outbreaks of infection in captive bearded dragons (Pogona barbata) (1, 2). Members of the genus *Nannizziopsis* are capable of infecting a wide range of reptile hosts and are a leading cause of often fatal fungal dermatitis in captive animals, sometimes referred to as “yellow fungus disease” (3). *N. barbatae* has become increasingly prevalent in a free-living population of eastern water dragons (Intellagama lesueurii), presenting an emergent threat to populations of wild Australian reptiles.

The isolate of *N. barbatae* was obtained via a skin swab taken from a lesion from an infected dragon, selected using Mycosel agar (Becton, Dickinson), and cultivated further on potato dextrose agar for 2 weeks at 25°C. Genomic DNA for Illumina short-read sequencing was extracted from mycelium from a pure plate culture using the DNeasy blood and tissue kit (Qiagen) following the manufacturer’s protocol for cultured cells. A single DNA library was sequenced on the HiSeq platform, generating 221 million 150-bp paired-end reads, which were trimmed and filtered using Trimmomatic v0.39 (4).

High-molecular-weight DNA was extracted for Nanopore sequencing from mycelium from the same isolate using a modified protocol based on Mayjonade et al. (5), which is described in detail on Protocols.io (6). Between 1 and 2 g of mycelium was homogenized and purified using a sorbitol wash solution described by Inglis et al. (7). DNA was size selected for fragments of ≥20 kb using a PippinHT instrument (Sage Science).

A long-read DNA sequencing library was prepared according to the Oxford Nanopore Technologies protocol 1D genomic DNA by ligation (SQK-LSK109). Sequencing was performed on a MinION Mk1B device using a FLO-MIN106 R9.4.1 revD flow cell according to the manufacturer’s instructions. Base calling of MinION fast5 reads to FASTQ format was performed with Guppy software v4.0.14 (Oxford Nanopore Technologies). Sequencing yielded 147,481 reads with an *N*_50_ value of 32.95 kb (read lengths up to 182 kb), which totaled 3.46 Gbp (approximately 110× coverage). Next, the read data were analyzed and filtered using two NanoPack tools (8); quality was inspected with NanoPlot v1.28.2, and long reads were processed with NanoFilt v2.7.1 using the parameters -l 5000 -q 7 --headcrop 200 --tailcrop 200.

Illumina short reads and Nanopore long reads were assembled simultaneously with MaSuRCA v3.4.1 (9) using default parameters except for allowing up to 35× coverage of Nanopore long reads. The resulting *de novo* assembly of 11 contigs was 31.54 Mb in total length with a contig *N*_50_ value of 6.19 Mb and a GC content of 40.36%. The largest contig was 9.29 Mb in size. Completeness of the assembly was assessed using the BUSCO v4.0.6 (10) tool against the ascomycota_odb10 database of 1,706 genes. This resulted in the identification of 97.8% complete and single-copy genes. Illumina short-read data mapped to the assembly with a 99.87% overall alignment rate and with 89.19% aligning uniquely in pairs. Telomeric repeats (TTAGGG_N_) were identified using the FindTelomeres script (11) at one end of 3 of the contigs and at both ends of contig 5. The karyotype of this species is currently unknown; however, at least four chromosomes were identified in the haploid *Coccidioides* spp. (12–14), fungal pathogens also from the order *Onygenales*. The assembly was masked for repetitive regions (12.24% of the genome) using RepeatMasker v4.1.0 (15) with default settings and using a custom repeat database built from the draft assembly using RepeatModeler2 v2.0.1 (16), also with default settings.

### Data availability.

This whole-genome shotgun project has been deposited at DDBJ/ENA/GenBank under the accession number JACVYH000000000. The version described in this paper is version JACVYH010000000. The sequence reads were deposited in association with BioProject PRJNA662660.
